# Synthesis and characterization of magnetic biochar adsorbents for the removal of Cr(VI) and Acid orange 7 dye from aqueous solution

**DOI:** 10.1007/s11356-020-09275-1

**Published:** 2020-06-10

**Authors:** Chella Santhosh, Ehsan Daneshvar, Kumud Malika Tripathi, Pranas Baltrėnas, TaeYoung Kim, Edita Baltrėnaitė, Amit Bhatnagar

**Affiliations:** 1 Department of Environmental and Biological Sciences, University of Eastern Finland, P.O. Box 1627, FI-70211 Kuopio, Finland; 2 Department of Electronics and Communication Engineering, Koneru Lakshmaiah Education Foundation, Vaddeswaram, AP India; 3 Department of Bionanotechnology, Gachon University, 1342 Seongnamdaero, Seongnam, 13120 South Korea; 4 Institute of Environmental Protection, Vilnius Gediminas Technical University, Saulėtekio al. 11, 40 Vilnius, Lithuania

**Keywords:** Biochar, Magnetic nanocomposites, Cr(VI), Acid orange 7 dye, Adsorption, Reusability

## Abstract

In this study, different types of magnetic biochar nanocomposites were synthesized using the co-precipitation method. Two biochar materials, namely, sewage sludge biochar and woodchips biochar, were prepared at two different temperatures, viz., 450 and 700 °C. These biochars were further modified with magnetic nanoparticles (Fe_3_O_4_). The modified biochar nanocomposites were characterized using field emission–scanning electron microscopy (FE-SEM), X-ray diffraction (XRD), transmission electron microscopy (TEM), Brunauer–Emmett–Teller (BET), SQUID analysis, X-ray photoelectron spectroscopy (XPS), and Fourier-transform infrared spectroscopy (FTIR). The potential of prepared adsorbents was examined for the removal of hexavalent chromium (Cr(VI)) and Acid orange 7 (AO7) dye from water as a function of various parameters, namely, contact time, pH of solution, amount of adsorbents, and initial concentrations of adsorbates. Various kinetic and isotherm models were tested to discuss and interpret the adsorption mechanisms. The maximum adsorption capacities of modified biochars were found as 80.96 and 110.27 mg g^-1^ for Cr(VI) and AO7, respectively. Magnetic biochars showed high pollutant removal efficiency after 5 cycles of adsorption/desorption. The results of this study revealed that the prepared adsorbents can be successfully used for multiple cycles to remove Cr(VI) and AO7 from water.

**Electronic supplementary material:**

The online version of this article (10.1007/s11356-020-09275-1) contains supplementary material, which is available to authorized users.

**Figure:**
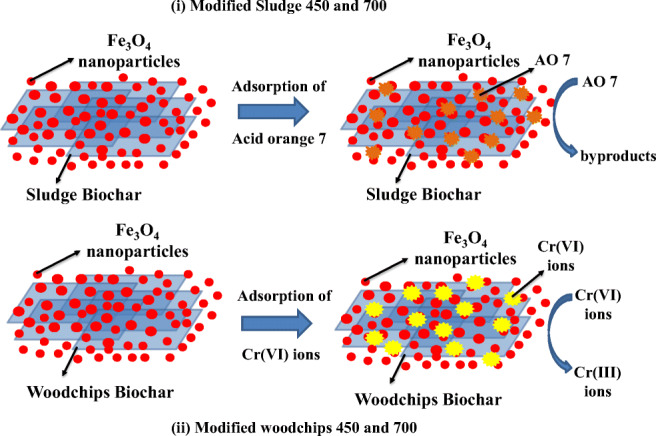
Graphical Abstract

## Introduction

Presence of elevated concentrations of inorganic and organic contaminants in water bodies is considered a serious global issue. Heavy metals and dyes are discharged to the aquatic environment through effluents of various industries such as mining, textile, dyeing and tanning, and electroplating (Yin et al. 2008). Metals have high solubility in water, and due to their low biodegradability, these can be accumulated in the environment and microorganisms and further transported to human body (Sherlala et al. 2018). Exposure to heavy metal ions, especially As(III)/(As(V)), Hg(II), Cd(II), Pb(II), and Cr(VI), even at lower concentrations, can have detrimental effects on human health, such as different types of cancers, and neurological and mental problems (Xu et al. 2018).

Another important source of water contamination is synthetic dyes, which are classified as organic pollutants. Dyes are complex aromatic molecules that are highly water soluble and resistant for biodegradation. These organic colorants are the first visible pollutants in water. The coloration of water due to dye effluents prevents the sunlight penetration into the aquatic ecosystems and disturbs the photosynthesis process (Daneshvar et al. 2013; Daneshvar et al. 2014). Due to the high toxicity, non-biodegradability, and bio-accumulation, wastewater containing synthetic dyes and heavy metal ions must be treated before discharging it into the nature.

Several physicochemical and biological methods, including electrochemical, filtration, reverse osmosis, ion exchange, chemical precipitation, adsorption, and coagulation, have been used for the ouster of dyes and heavy metals from water (Fu and Wang 2011). Among the existing methods, adsorption is particularly attractive for the removal of organic (such as dyes) and inorganic (such as heavy metals) pollutants from wastewater. Adsorption is an inexpensive, eco-friendly, and efficient method for the removal of many pollutants from contaminated water. Various locally available minerals, and organic and biological materials such as agricultural wastes have been used as adsorbents (Thines et al. 2017).

Carbon-based adsorbents have been found to be the favorite choice for water treatment because of their distinctive chemical and physical properties. Though activated carbon as a commercial adsorbent has high sorption capacity, sometimes the high cost and lengthy synthesis procedure limit its practical applications (Daneshvar et al. 2017). There is an increasing interest in exploration of sustainable materials for water remediation. Biochar is produced by the carbonization of biomass in an oxygen-limited atmosphere (Lehmann et al. 2006). As a low-cost material, it can be a suitable alternative to expensive activated carbon for removing organic pollutants and heavy metal ions from water. High porosity, physiochemical stability in water, and natural abundance of precursors make biochar a promising candidate for water remediation. In addition, carboxyl (–COOH) and hydroxyl (–OH) functional groups on the surface of biochar act as sorption sites for the toxic metal ions. In addition, surface complexation and/or ion exchange are considered the main mechanisms of sorption by biochar (Wan et al. 2018).

Though micro- or nanoparticles of biochar exhibit extreme adsorption capacity due to high surface area, the residual powder of biochar can cause secondary pollution in water. Hence, an efficient method is necessary to separate biochar from water after the adsorption process. This problem can be resolved by synthesizing magnetic biochar that can be separated easily from water with the use of an external magnetic field (Wang et al. 2018). Synthesis of composites as efficient adsorbents has got attention in water treatment as the properties of two or more individual components (parent materials) are combined in one composite (Liang et al. 2018). Composites of magnetic biochar, because of their large specific surface area and high potential of recovery from water, have all of the required features of efficient adsorbents.

Literature review reveals that biochar can be derived from a variety of biomass such as micro- and macroalgal biomasses, sawdust, agricultural wastes, fruit peels, wood, and wastewater sludge (Son et al. 2018). Wood chips and sludge from wastewater treatment plant have been found to be a potentially suitable source for biochar production due to their low-cost and free availability. Huge volumes of wood chips are produced during construction and demolition activities in Finland (Husgafvel et al. 2017) and other EU countries (Jonsson and Rinaldi 2017).

In this study, biochars were synthesized from wood chips and sewage sludge at two different temperatures, viz., 450 and 700 °C, to remove Cr(VI) (inorganic) and AO7 (organic) pollutants from water. The main aim of this study was to compare removal efficiencies of two important classes of aquatic pollutants, viz., Cr(VI) and AO7 dye, by un-modified and modified (magnetic) biochars. The effects of different variables, viz., initial solution pH, biochar dose, metal or dye concentration, and contact time on the adsorption capacity have been studied. Isotherms and kinetics of adsorption processes were studied to elucidate the mechanism. Furthermore, the structure and morphology of the adsorbents were analyzed by various spectroscopic and microscopic techniques.

## Experimental section

### Materials

Analytical-grade K_2_Cr_2_O_7_ (≥ 99.0%), Orange II sodium salt (AO7 dye) (dye content ≥ 85 %), ferrous chloride (FeCl_2_.4H_2_O) (≥ 99.0%), sodium hydroxide (NaOH) (≥ 98%), and ferric chloride (FeCl_3_·6H_2_O) (97%) were purchased from Sigma-Aldrich. All the solutions were prepared in distilled water.

### Preparation of modified (magnetic) biochar

Biochar samples of sludge and woodchips were prepared at 450 and 700 °C (Baltrėnaitė et al. 2017). The prepared biochars were labelled as S-450, S-700, WC-450, and WC-700. Magnetic biochar samples were synthesized using the co-precipitation method. Typically, 100 mL of deionized (DI) water was added to a conical flask of 250 mL containing ferric chloride and ferrous chloride. The mixture was agitated on a magnetic stirring for 1 h. Later, 1 g of biochar sample was added to the DI water, followed by further stirring for 30 min. Simultaneously, another conical flask of 250 mL containing DI water (100 mL) with an appropriate amount of NaOH was stirred for 1 h at 80 °C. The above solutions were mixed in one beaker and stirred for 2 h at 100 °C. After 2 h, the magnetic biochars were accumulated using an external magnetic field. The collected modified biochars were washed with ethanol and then water for several times. Finally, the synthesized biochars were dried in a vacuum oven at 45 °C overnight. Modified biochar samples were denoted as MS-450, MS-700, MWC-450, and MWC-700.

### Batch adsorption studies

The stock solutions of Cr(VI) and AO7 were prepared with analytical grade of K_2_Cr_2_O_7_ and AO7 dye salts. The adsorption studies of Cr(VI) and AO7 dye with synthesized biochar composites were carried out in centrifuge tubes of polyethylene by batch method containing adsorbate solutions of known concentrations and a desired amount of modified biochar adsorbent at a speed of 80 rpm on a roller shaker. Contact time (0–200 min.), initial adsorbate concentration (5–100 mg L^−1^), adsorbent dosage (0.5–2 g L^−1^), and solution pH (2–10) were studied as variable parameters. The solution pH was adjusted by adding 0.1 M HCl or NaOH solutions in a negligible amount. While studying a single parameter during the experiment, the rest of the parameters were kept constant. The solid and liquid phases were separated after shaking for predetermined time by external magnetic field, followed by filtration with cellulose nitrate membrane filters of 0.45 μm. The concentrations of AO7 dye and Cr(VI) (1,5-diphenylcarbohydrazide method) were measured using UV–visible spectrophotometer (UV-2401PC) at the maximum absorbance wavelength of 485 nm and 540 nm, respectively.

The removal efficiency of Cr(VI) ions and AO7 dye onto the synthesized modified biochar was calculated referring to the difference among the initial (C_i_) and equilibrium (C_e_) concentrations of pollutants in the liquid phase after the filtration. The removal efficiency of pollutants and adsorption capacity of biochars were calculated by the following equations:

1 $$ \mathrm{Adsorption}\left(\%\right)=\frac{C_{\mathrm{i}}-{C}_{\mathrm{e}}}{C_{\mathrm{i}}}\times 100 $$

2 $$ {q}_{\mathrm{e}}=\frac{v\left({C}_{\mathrm{i}}-{C}_{\mathrm{e}}\right)}{W} $$

where *C*_i_ and *C*_e_ are the initial and equilibrium dye and/or metal ions concentration; *C*_e_ and *q*_e_ are concentration of the dye and metal ions and equilibrium adsorption capacity at equilibrium; *W* is the weight of adsorbent in grams (g) and *v* is the volume of dye and metal ion solution (L).

### Reusability experiment

For the reusability studies, pollutant-loaded materials (0.005 g) in 10 mL of NaOH (0.1 M) were shaken at 80 rpm for 2 h at room temperature. Using external magnetic field, the adsorbent was separated and the adsorbate was further filtered with the 0.45 μm membrane filter. Final solution was analyzed for remaining metal or dye concentration by using UV technique. The obtained metal or dye desorbed materials were further used and the process was repeated up to five cycles (adsorption–desorption) to check the reusability potential of the prepared materials.

### Measurements and characterization

The functional groups on the surface of adsorbents were analyzed by using Fourier-transform infrared (FT-IR) spectral analysis with Perkin Elmer (Version 10.5.1) in solid state with KBr. Analysis of the phase purity and crystalline nature of the samples has been performed with a Rigaku RINT-2000 X-ray diffractometer, endowed with a Cu K_α_ radiation generator. Surface charge of the samples was measured with PALS zeta potential analyzer (Brookhaven instruments) in aqueous solution at neutral pH. XPS analysis was performed on a ULVAC-PHI X-tool XPS spectrometer with an excitation source of Al K_α_. The size and morphology of the prepared raw and modified biochar materials were determined with field emission–scanning electron microscope (FE-SEM) S-4700, HITACHI, Japan, with an accelerating voltage of 20 kV. High-resolution transmission electron microscopy (HR-TEM) images were obtained using a FEI Tecnai G2 F30 transmission electron microscope with a field emission gun operated at 300 kV. In order to prepare the TEM/HR-TEM samples, biochar was first dispersed in water via sonication for 15 min and then a small drop of the suspension was cast on a carbon-film-coated copper grids and dried overnight in a vacuum oven. The magnetic properties of the samples were acquired by SQUID magnetometer (Quantum Design MPMS3) at room temperature and constant external applied field of 5 kOe using powder of the samples.

## Results and discussion

### XRD analysis

**Fig. 1:**
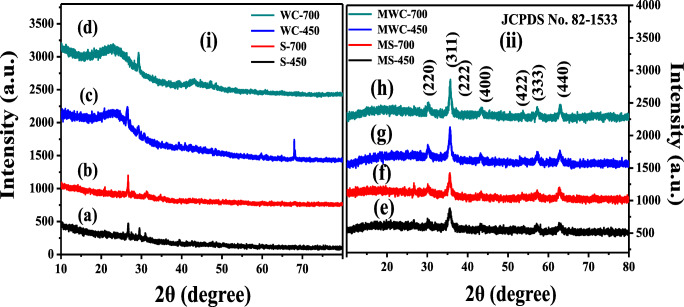
XRD analysis of un-modified S-450 (a), un-modified S-700 (b), un-modified WC-450 (c), un-modified WC-700 (d), modified S-450 (e), modified S-700 (f), modified WC-450 (g), modified WC-700 (h)

Figure 1 shows the diffraction patterns of both un-modified and modified biochar materials which were obtained at 450 °C and 700 °C. A broad peak was observed at the 2θ value of around 22° for both WC-450 and WC-700. Figure 1(i) (a, b) shows that both amorphous and crystalline structures, and presence of some inorganic minerals, such as SiO_2_ and Al/Si oxides in the sewage sludge biochar, with the typical 21.22°, 26.44°, 28.32°, and 39.45° in the 2θ degree (Zhou et al. 2017). The peak at 22° was ascribed to crystallinity of cellulose, which was shown in Fig. 1(i) (c, d). Similar XRD results were reported by Shaaban et al. (2013) for raw rubber wood sawdust (RWSD) and (Wang et al. 2009) for raw woodchips. Figure 1(ii) shows the XRD patterns for modified biochar materials. All the crystal planes (220), (311), (222), (400), (422), (333), and (440) with respect to 2θ values of 30.20°, 35.69°, 36.92°, 42.98°, 52.93°, 57.23°, and 62.91°, respectively, were matched with the standard JCPDS No. 82-1533, which reveals that the biochar materials were modified successfully—coated with Fe_3_O_4_ nanoparticles without any other morphological impurities.

### Morphological analysis

**Fig. 2:**
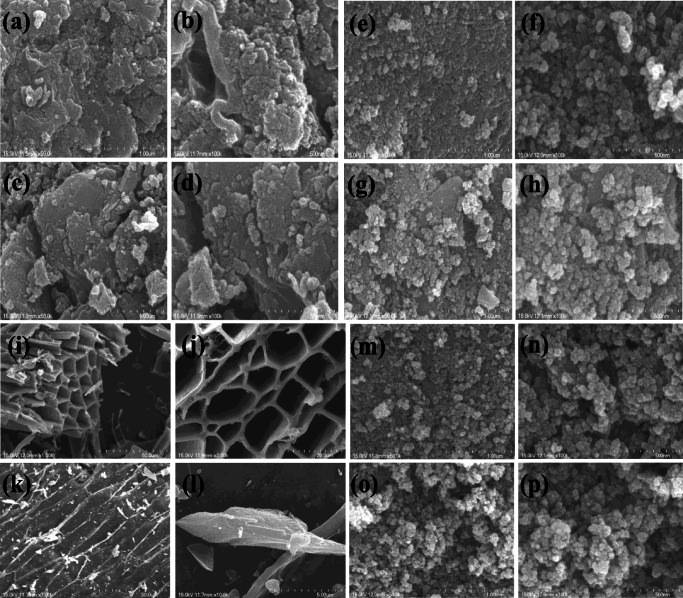
SEM images of un-modified S-450 (a, b), un-modified S-700 (c, d), modified S-450 (e, f), modified S-700 (g, h), un-modified WC-450 (i, j), un-modified WC-700 (k, l), modified WC-450 (m, n), modified WC-700 (o, p)

Figure 2 shows the SEM images of both un-modified and modified biochar materials of S-450 and S-700. Figure 2 (a, b) shows SEM images of the un-modified S-450, and Fig. 2 (c, d) shows the SEM images of un-modified biochar of S-700. The SEM images clearly show that the biochar materials exhibit flake like morphology, where size varied in micrometer ranges. Figure 2 (e–h) shows the SEM images of modified biochar S-450 and S-700, where the flake-like structures are decorated with Fe_3_O_4_ nanoparticles, and the nanoparticles were agglomerated. The average particle size of Fe_3_O_4_ nanoparticles is in the range of 50–100 nm. Figure 2 (i, j) shows the un-modified biochar materials of WC-450, and Fig. 2 (k, l) shows the un-modified biochar materials of WC-700. WC-450 (Fig. 2 (i, j)) clearly shows the hollow structures which are in μm sizes, but WC-700 (Fig. 2 (k, l)) do not show any hollow structures because the woodchips were sintered at 700 °C and became more amorphous in nature and the same was confirmed by the XRD pattern, whereas Fig. 2 (m–p) shows the modified biochar WC-450 and WC-700, and herein, the hollow structures were fully decorated with Fe_3_O_4_ nanoparticles, and the nanoparticles were agglomerated. The average particle sizes of Fe_3_O_4_ nanoparticles were in the range of 50–100 nm.

**Fig. 3:**
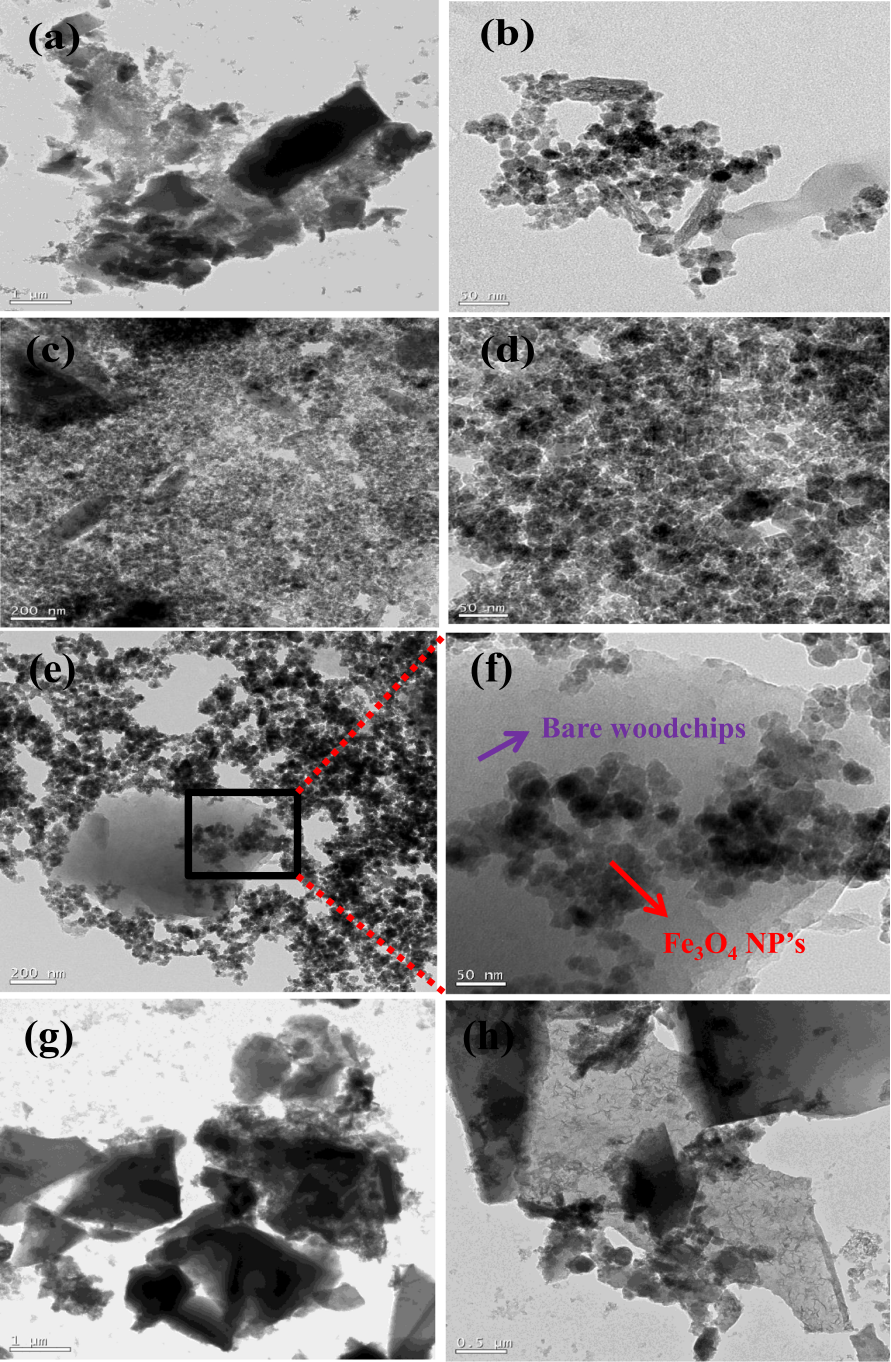
TEM images of modified S-450 (a, b), modified S-700 (c, d), modified WC-450 (e, f), modified WC-700 (g, h)

Figure 3 (a–d) shows the TEM images of modified S-450 and S-700. From the figure, it can be observed that the magnetic (Fe_3_O_4_) nanoparticles aggregated and look rugged on the surface of the sludge biochar materials. As clearly seen in the figure, flake-like structure is observed in sludge biochar doped with Fe_3_O_4_ nanoparticles. The average crystalline diameters of Fe_3_O_4_ nanoparticles are in range of ~ 15–25 nm. Figure 3 (e–h) shows the TEM images of modified WC-450 and WC-700. It can be clearly seen that Fe_3_O_4_ nanoparticles decorated the surface of woodchip biochar materials. Hence, it can be concluded that Fe_3_O_4_ nanoparticles were not only present on the surface of biochar but also surrounded on the biochar.

### XPS and FT-IR analysis

**Fig. 4:**
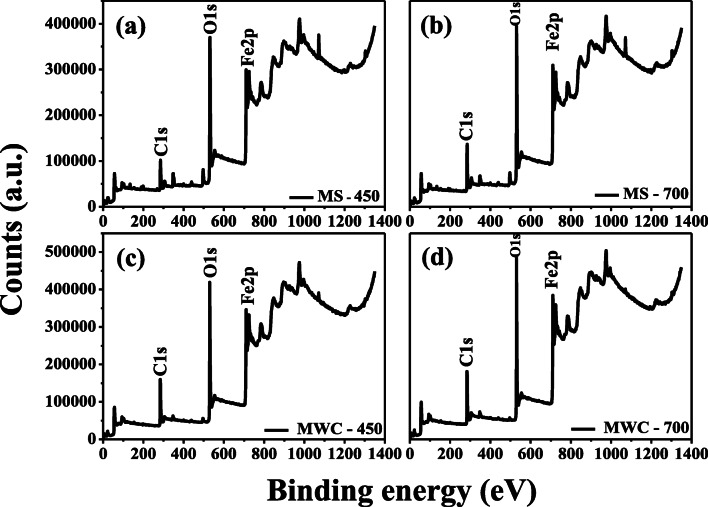
Full scan survey of modified S-450 (a), modified S-700 (b), modified WC-450 (c), modified WC-700 (d)

**Fig. 5:**
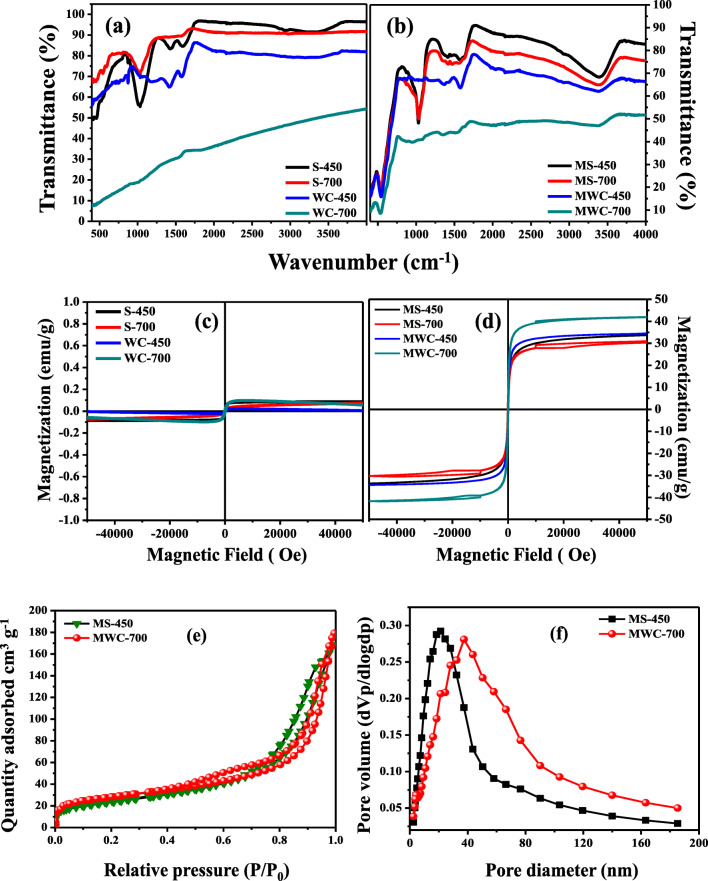
FT-IR analysis of un-modified biochar materials (a), modified biochar materials (b), magnetic hysteresis (M-H) studies of un-modified biochar materials (c), modified biochar materials (d), nitrogen adsorption–desorption isotherm (e), BJH adsorption pore size distribution curve (f) of modified S-450 and modified WC-700

Figure 4 shows the full scan X-ray photoelectron spectroscopy (XPS) spectra of modified biochar sludge and woodchips. The XPS spectrum in Fig. 4 reveals that both the samples are basically composed of C, Fe, and O core-elements. The ratios of C, O, and Fe in MS-450 and MS-700 were 46.48%, 22.72%, and 15.8%, and 29.75%, 42.56%, and 15.28%, respectively. On the other hand, in MWC-450 and MWC 700, the ratios of C, O, and Fe were 36.1%, 40.32%, and 19.01% and 47. 36%, 39%, and 18.91%, respectively. The XPS results indicated that modification with Fe_2_O_3_ nanoparticles in biochar materials was effective with a favorable quantity of Fe. Figure 5 (a, b) shows the FT-IR spectra of both, the un-modified and modified biochar materials, respectively. The main peaks that represent the vibration of the functional groups in the un-modified biochar materials were as follows: –OH—3456 cm^−1^; aromatic C = C and C = O—1595 and 1698 cm^−1^; and C–O–C—1048 cm^−1^. Comparison of the spectra of the un-modified biochar materials and modified biochar materials reveals that the strong absorption peak at 562 cm^−1^ is caused by Fe–O of iron oxide. This indirectly confirmed the presence of Fe_3_O_4_ in the modified biochar materials.

### Magnetic, zeta potential, and BET surface area studies

For recycling and easy recovery from the aqueous solution, magnetic sorbents could be worth enough for water remediation. The magnetic hysteresis of prepared modified biochar and un-modified biochar materials was measured at the magnetic fields of − 50,000 ≤ H ≤ 50,000 Oe at room temperature. Figure 5 (c, d) shows the magnetic hysteresis of both the un-modified and modified biochar materials. It can be clearly seen from the figure that the un-modified biochar materials have almost zero magnetization, while modified biochar materials show significant magnetization values. This is because of the presence of Fe_3_O_4_ nanoparticles on the surface of biochar materials. The magnetization of modified biochar materials was 33.8, 30.3, 34.49, and 41.87 emu g^−1^ for MS-450, MS-700, MWC-450, and MWC-700, respectively. Fe_3_O_4_ nanoparticles decorating the biochar surface behave magnetically active, which in turn affect the magnetic properties of the biochars, that was also confirmed by TEM and SEM images. In addition, the zeta potential measurement values also showed that the zeta potential of the modified biochar materials can be reduced by the introduction of Fe_3_O_4_ nanoparticles. The zeta potentials of the un-modified and modified biochar materials are presented in **Table S1****(Supplementary Information)**.

The BET specific surface area isotherm of the modified biochar materials (MS-450 and MWC-700) is shown in Fig. 5 (e). The isotherm was ascribed to H2-type hysteresis loop and type IV shape. The specific surface areas of MS-450 and MWC-700 were 127.98 and 99.83 m^2^ g^−1^, respectively. In addition to this, the pore distribution curve is also shown in Fig. 5 (f) by using Barrett–Joyner–Halenda (BJH) method. The pore diameter of MS-450 and MWC-700 was 21.3 and 37.4 nm, respectively. The results showed that the prepared modified biochar materials possessed the mesoporous surface with high specific surface area.

### Adsorption studies

#### Comparison of un-modified and modified biochar

The comparison results for both un-modified and modified biochar materials for the adsorption of Cr(VI) and AO7 dye are presented in **Fig. S1****(Supplementary Information)**. As seen from Fig. S1, AO7 dye was removed well by MS-450 and MS-700, whereas Cr(VI) was well adsorbed by MWC-450 and MWC-700. This difference in sorption capacity of AO7 dye and Cr(VI) on different biochars might be due to several reasons, for example, differences in the chemical structure and molecular weight of each species. Both Cr(VI) and AO7 dye were well adsorbed by the modified biochar materials as compared with the un-modified biochar materials. So, all the parameters were tested only on the modified sludge biochar and modified woodchips biochar.

#### Effect of contact time

**Fig. 6:**
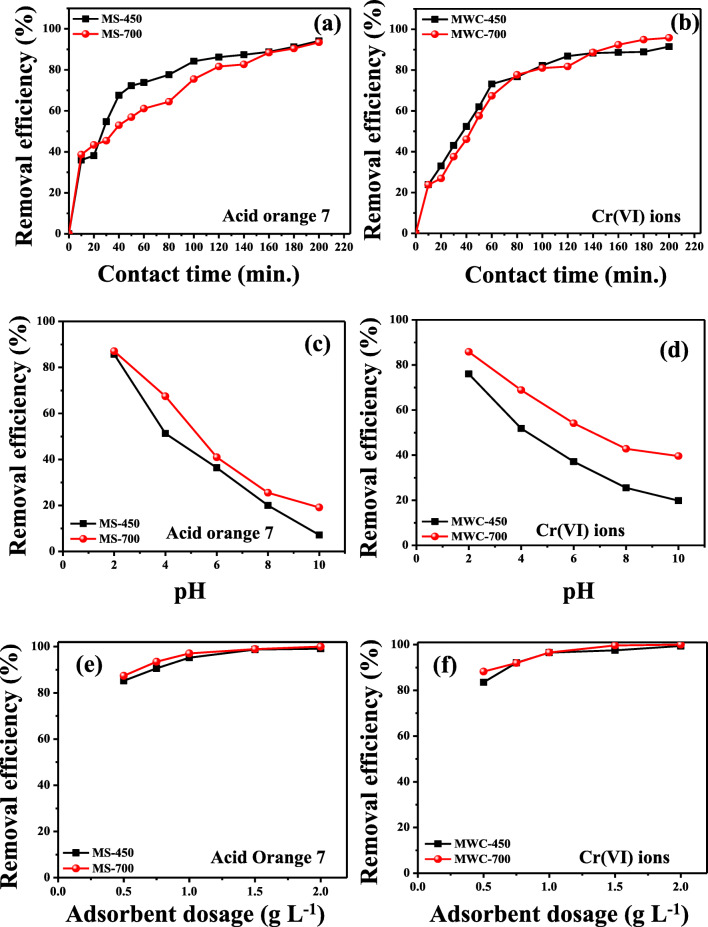
Effect of contact time (a, b) on AO7 dye and Cr(VI) removal onto modified biochar materials (initial concentration of 10 mg L^−1^, pH 2, adsorbent dosage 0.5 g L^−1^), effect of pH (c, d) on AO7 dye and Cr(VI) removal onto modified biochar materials (initial concentration 10 mg L^-1^, adsorbent dosage 0.5 g L^-1^, contact time 180 min), effect of adsorbent dosage (e, f) on AO7 dye and Cr(VI) removal onto modified biochar materials (initial concentration 10 mg L^−1^, pH 2, contact time 180 min)

The effect of contact time on the removal efficiency of AO7 dye and Cr(VI) ions by modified sludge and woodchips, respectively, was examined at various time intervals up to 200 min. Figure 6 (a) shows that the adsorption of AO7 dye was rapidly increased during the initial 10 min and the removal efficiency was reached almost 40%. Afterwards, the adsorption rate gradually decreased until the acquisition of equilibrium around 160 min with the maximum removal efficiency of 90%. However, MS-450 showed a different pattern of AO7 adsorption as compared with MS-700, but the equilibrium time of both adsorbates was 160 min. Figure 6 (b) shows that 20% of Cr(VI) was removed rapidly within 10 min. Afterwards, the rate of adsorption slowed down until it reached the equilibrium around 140 min with the maximum removal efficiency of 95%. The relatively fast adsorption of AO7 dye and Cr(VI) onto the modified biochar materials was probably due to the strong attraction between the pollutants and a large number of binding sites originating from the surface of the modified biochar materials. Compared with the initial stages, the adsorption was slower because of occupying the available binding sites on the surface of modified biochars. Hence, the results showed that the equilibrium time was around 180 min for both pollutants.

#### Effect of pH

In the adsorption process, the initial pH of aqueous medium is quite significant as compared with other parameters as it can affect the properties of both adsorbent and adsorbate. Figure 6 (c, d) shows the effect of pH on the adsorption of AO7 dye and Cr(VI) ions onto the modified biochar adsorbents. Figure 6 (c) depicts that the adsorption of AO7 dye was better at acidic pH compared with the basic pH. As pH increased from 2.0 to 10, the removal efficiency of AO7 dye onto the prepared modified sludge adsorbent was gradually decreased from 90 to 10%. Figure 6 (d) shows the adsorption of Cr(VI) ions onto the prepared modified woodchip adsorbents; the removal efficiency was higher at acidic pH of 2.0, but it decreased when pH increased. The speciation of Cr(VI) will be changed according to the pH of the aqueous solution. Various forms of Cr(VI) that exist include HCrO_4_^−^, CrO_4_^2−^, and Cr_2_O_7_^2−^. Based on the pH value, various forms of Cr(VI) are predominant. HCrO_4_^−^ and Cr_2_O_7_^2−^ are predominant at pH of 2.0–6.0, while CrO_4_^2−^ is predominant at pH values > 6.0 (Mohan and Pittman 2006). At higher pH values > 6.0, the protonation of the modified biochar adsorbent is in a weak state to generate electrostatic repulsion between the negatively charged ions and the surface of the prepared adsorbents (Yang et al. 2017). At low pH values from 2.0 to 6.0, HCrO_4_^−^ is more predominant, which is the main form to remove Cr(VI) ions from the aqueous solution. Therefore, at lower pH values, the removal efficiency was higher than at higher pH values (Mohan and Pittman 2006). Thus, the prepared modified biochar adsorbents exhibited maximum removal efficiency of AO7 dye and Cr(VI) ions at pH of 2.0. Hence, pH 2.0 was chosen for the study.

#### Effect of sorbent dosage

Sorbent dosage is another significant parameter because it deals with the adsorbent–adsorbate interaction and shows the equilibrium of the system. The removal percentages of AO7 and Cr(VI) were studied in response to different adsorbent dosage from 0.5 to 2.0 g L^−1^ (Fig. 6 (e, f)). The figure shows that the removal efficiency of AO7 dye and Cr(VI) ions increased as the dosage of biochar was increased. The enhancement of removal efficiency with higher adsorbent dose is attributed to the availability of more binding sites for pollutant adsorption. However, at a certain dosage (1 g L^−1^), both the pollutants reached the saturation point. This may be due to the sufficient adsorbent dosage for a certain concentration of pollutants (10 mg L^−1^) in the solution. Hence, the highest removal efficiencies of AO7 dye and Cr(VI) ions were achieved as 99.8% and 100%, respectively, with 1.0 g L^−1^ of the modified biochar adsorbents.

#### Kinetic studies

**Figure S2****(Supplementary Information)** shows the kinetic graph of AO7 dye and Cr(VI) removal onto modified biochar by plotting time (min) versus q_e_ (mg g^-1^). The adsorption kinetics of pollutants revealed that there is a gradual increase in the adsorption of pollutants with time and after ca. 180 min, the equilibrium was achieved (seen by almost constant plateau region in the graph) for modified biochar adsorbents. The obtained graphs suggested the equilibrium time of 180 min for the adsorption of AO7 and Cr(VI) by modified biochars.

In order to better understand the rate-controlling steps, the adsorption kinetics of selected pollutants onto the prepared modified biochar adsorbents was studied. Various non-linear forms of kinetic models, viz., pseudo-first-order (Lagergren 1898), pseudo-second-order (Ho and McKay 1999), Avrami (Avrami 1939), and intra-particle diffusion (Weber and Morris 1963) models (Eqs. 3–6) were used to study the kinetics of the process.

3 $$ {q}_t={q}_{\mathrm{e}}\left(1-{e}^{-{k}_1t}\right) $$

4 $$ {q}_t=\frac{k_2{q}_e^2t}{1+{k}_2{q}_{\mathrm{e}}t} $$

5 $$ {q}_t={q}_{\mathrm{e}}\left(1-{e}^{\left(-{\left({K}_{\mathrm{AV}}t\right)}^n AV\right)}\right) $$

6 $$ {q}_t={K}_{\mathrm{p}}{t}^{\frac{1}{2}}+I $$

where *q*_e_ is the adsorption capacity of pollutant ions at equilibrium and *q*_t_ (mg g^−1^) is the adsorption capacity of pollutants at time *t* (min), respectively; *k*_1_ (min^−1^) is the pseudo-first-order rate constant and *k*_2_ (g mg^−1^ min^−1^) is the pseudo-second-order rate constant. Avrami constant was denoted as *K*_AV_ (min^−1^), whereas *K*_p_ (mg g^−1^ min^−1/2^) is the intra-particle diffusion constant and *I* (mg g^−1^) is the intercept. The experimental data was fitted into different kinetic models (mentioned above), and the results are shown in **Fig. S2****(a) and (c)** for AO7 dye and **Fig. S2****(b) and (d)** for Cr(VI) ions onto modified sludge (MS-450 and MS-700) and woodchips (MWC-450 and MWC-700) biochars, respectively. Tables 1 and 2 show kinetic parameters for AO7 dye and Cr(VI) adsorption onto modified biochars. The theoretical (*q*_e_) values of selected pollutants were close to the obtained experimental values with minimum and maximum RMSE, and correlation coefficient (*R*^2^), respectively for the pseudo-second-order kinetic model for modified biochar adsorbents. Based on the above analysis, pseudo-second-order model was found to be best fitted model as compared with the pseudo-first-order and Avrami models.

**Table 1:** Kinetic parameters for AO7 dye onto MS-450 and MS-700

Adsorbent	Pseudo-second-order model					
	*q*_e(exp)_ (mg g^−1^)	*k*_2_ (min^−1^)		*q*_e(cal)_ (mg g^−1^)	RMSE	*R*^2^
MS-450	13.29	0.014		12.79	3.12	0.990
MS-700	16.58	0.0010		20.18	4.26	0.992
Adsorbent	Avrami model					
	*q*_e(exp)_ (mg g^−1^)	*K*_av_	*n*_av_	*q*_e(cal)_ (mg g^−1^)	RMSE	*R*^2^
MS-450	13.29	1.84	6.80	11.87	3.22	0.982
MS-700	16.58	0.0042	4.45	16.47	4.43	0.872
Adsorbent	Intra-particle diffusion model					
	*C*_i_ (mg L^−1^)	*I* (mg g^−1^)		*K*_p_ (mg g^−1 min-0.5^)	RMSE	*R*^2^
MS-450	10	5.24		0.25	4.81	0.886
MS-700	10	3.32		0.95	5.43	0.986

**Table 2:** Kinetic parameters for Cr(VI) onto MWC-450 and MWC-700

Adsorbent	Pseudo-second-order model					
	*q*_e(exp)_ (mg g^−1^)	*k*_2_ (min^−1^)	*q*_e(cal)_ (mg g^−1^)	RMSE		*R*^2^
MWC-450	20.74	0.001	20.98	7.94		0.980
MWC-700	19.98	0.010	22.18	4.26		0.992
Adsorbent	Avrami model					
	*q*_e(exp)_ (mg g^−1^)	*K*_av_	*n*_av_	*q*_e(cal)_ (mg g^−1^)	RMSE	*R*^2^
MWC-450	20.74	0.033	0.65	25.54	8.10	0.830
MWC-700	19.98	0.042	1.45	15.88	4.43	0.872
Adsorbent	Intra-particle diffusion model					
	*C*_i_ (mg L^−1^)	*I* (mg g^−1^)		*K*_p_ (mg g^−1 min-0.5^)	RMSE	*R*^2^
MWC-450	10	0.93		1.61	8.90	0.980
MWC-700	10	3.32		0.95	5.43	0.986

#### Adsorption isotherms

In this work, Freundlich, Langmuir, Redlich–Peterson, and Sips isotherm models have been studied to describe equilibrium data of Cr(VI) and AO7 adsorption onto modified biochars. Monolayer and multilayer adsorptions onto different surfaces (homo- and heterogenous) of the adsorbents were analyzed by Langmuir (Langmuir 1918) and Freundlich (Freundlich 1907) isotherm models, respectively. The combination of formerly mentioned models gives the Sips model with three parameters (Sips 1948). Also, the adsorption can be pertained either in homo- or heterogenous systems leading to the Redlich–Peterson model, another three-parameter isotherm model (Redlich and Peterson 1959). The above mentioned non-linear isotherm models are presented by the Eqs. 7–10 as follows:

7 $$ {q}_{\mathrm{e}}=\frac{q_{\mathrm{m}{K}_{\mathrm{L}}{C}_{\mathrm{e}}}}{1+{K}_{\mathrm{L}}{C}_{\mathrm{e}}} $$

8 $$ {q}_{\mathrm{e}}={K}_{\mathrm{F}}{C}_{\mathrm{e}}^{\frac{1}{n}} $$

9 $$ {q}_{\mathrm{e}}=\frac{q_{\mathrm{m}{\left({K}_S{C}_{\mathrm{e}}\right)}^m}}{1+{\left({K}_S{C}_{\mathrm{e}}\right)}^m} $$

10 $$ {q}_{\mathrm{e}}=\frac{K_{\mathrm{RP}}{C}_{\mathrm{e}}}{1+{a}_{\mathrm{RP}}{C}_{\mathrm{e}}^{\beta }} $$

where *q*_e_ (mg g^−1^) is the amount of adsorbed Cr(VI) or AO7 per unit weight of biochars and *C*_e_ (mg L^−1^) is the equilibrium concentration of Cr(VI) or AO7; *q*_m_ is the maximum monolayer adsorption capacity of Langmuir model and *K*_L_ (L mg^−1^) is the Langmuir constant; *K*_F_ and *n* are the Freundlich constants and exponent, respectively; *K*_S_ (L mg^-1^) and *K*_RP_ (L g^−1^) and *a*_RP_ (L mg^-1^) are constants in Sips and Redlich–Peterson models, respectively. The equilibrium adsorption was examined based on the initial concentrations of ions from 5 to 100 mg L^−1^. Non-linear curves of isotherm models were obtained by plotting *C*_e_ versus *q*_e_ (**Fig. S3****, Supplementary Information**). Accordingly, isotherm parameters of each model related to AO7 and Cr(VI) adsorption are presented in Tables 3 and 4, respectively.

**Table 3:** Isotherm studies for AO7 dye onto MS-450 and MS-700

Adsorbent	Langmuir isotherm model			
	*q*_m_ (mg g^−1^)	*K*_L_	*R*_L_	*R*^2^
MS-450	110.27	0.053	0.003	0.987
MS-700	64.40	0.11	0.01	0.975
	Freundlich isotherm model			
	*K*_F_ (mg g^−1^) (L mg^−1^)^1/*n*^	1/*n*	-	*R*^2^
MS-450	19.94	0.35	-	0.696
MS-700	19.87	0.25	-	0.590
	Sips isotherm model			
	*q*_m_ (mg g^−1^)	*K*s (L mg^−1^)	*n*	*R*^2^
MS-450	167.39	0.11	0.52	0.945
MS-700	64.80	0.21	0.98	0.876
	Redlich–Peterson isotherm model			
	*K*_RP_ (L mg^−1^)	*q*_PR_ (mg g^−1^)	*n*	*R*^2^
MS-450	207.90	15.46	0.56	0.658
MS-700	207.91	18.73	0.72	0.503

**Table 4:** Isotherm studies for Cr(VI) onto MWC-450 and MWC-700

Adsorbent	Langmuir isotherm model			
	*q*_m_ (mg g^−1^)	*K*_L_	*R*_L_	*R*^2^
MWC-450	78.71	0.02	0.005	0.991
MWC-700	80.96	0.04	0.003	0.989
	Freundlich isotherm model			
	*K*_F_ (mg g^−1^) (L mg^−1^)^1/*n*^	1/*n*	-	*R*^2^
MWC-450	3.97	0.60	-	0.721
MWC-700	29.60	0.13	-	0.690
	Sips isotherm model			
	*q*_m_ (mg g^−1^)	*K*s (L mg^−1^)	*n*	*R*^2^
MWC-450	63.54	0.02	1.20	0.915
MWC-700	87.62	0.014	1.24	0.876
	Redlich–Peterson isotherm model			
	*K*_RP_ (L mg^−1^)	*q*_PR_ (mg g^−1^)	*n*	*R*^2^
MWC-450	154.29	3.98	0.39	0.754
MWC-700	197.80	8.91	0.53	0.848

Langmuir model, with the highest correlation coefficient (*R*^2^) from 0.975 to 0.991 (close to 1.00) (Tables 3 and 4), was selected to describe the fitness of isotherm to the experimental data. The Langmuir fitting suggests that AO7 and Cr(VI) adsorption took place onto homogenous surfaces of modified biochar materials in this study. The maximum monolayer adsorption capacities of AO7 and Cr(VI) onto modified sludge 450 and modified woodchips 700 were 110.27 mg g^−1^ and 80.96 mg g^−1^, respectively (Tables 3 and 4).

To compare the adsorption performance of synthesized biochars in this study with other adsorbents used for Cr(VI) and AO7 removal, a list of tested adsorbents and their maximum adsorption capacities are presented in Table 5. As it can be seen from the reviewed literature, MWC-700 showed higher adsorption capacity for Cr(VI) uptake as compared with other carbon- and biochar-based adsorbents. However, maximum adsorption capacity of MWC-700 was lower than clay- and silicon-based composites. It is also clear from Table 5 that MS-450 showed higher adsorption capacity of AO7 as compared with other reported adsorbents and it showed comparable adsorption capacity with activated carbon. High adsorption capacity of prepared adsorbents in this study confirmed that magnetic modification of woodchips and sewage sludge biochars can enhance their adsorption capacities for Cr(VI) and AO7 dye removal.

**Table 5:** Comparison of maximum adsorption capacities of various adsorbents for Cr(VI) and AO7 adsorption

Adsorbents	Maximum adsorption capacity (mg g^−1^)	Pollutants	References
Oxidized carbon	43.67	Cr(VI)	(AL Othman et al. 2013)
Shaddock peel–based activated carbon	50.76	Cr(VI)	(Tao et al. 2019)
Bentonite clay@MnFe_2_O_4_ composite	178.57	Cr(VI)	(Ahmadi et al. 2020)
Modified biochar–derived from corn stalks	23.07	Cr(VI)	(An et al. 2018)
Magnetic SiO_2_@CoFe_2_O_4_ nanoparticles	136.40	Cr(VI)	(Santhosh et al. 2017)
Activated carbon	109.05	AO7	(Li et al. 2017)
Treated algal biomass	71.05	AO7	(Kousha et al. 2012)
Inorganically modified mesoporous biochar	59.344	AO7	(Lin et al. 2020)
Modified multi-walled carbon nanotubes	59.52	AO7	(Jia et al. 2020)
Magnetic composites	48.12	AO7	(Yang et al. 2019)
MWC-450	78.71	Cr(VI)	This study
MWC-700	80.96	Cr(VI)	This study
MS-450	110.27	AO7	This study
MS-700	64.40	AO7	This study

Finally, *R*_L_ was calculated by Eq. (11) to determine whether the adsorption is favorable or not (Weber and Chakravorti 1974):

11 $$ {R}_{\mathrm{L}}=\frac{1}{1+{K}_{\mathrm{L}}{C}_i} $$

Different values of *R*_L_ are interpreted as follows: *R*_L_ = 0 (irreversible), 0 < *R*_L_ < 1 (favorable), *R*_L_ = 1 (linear), and *R*_L_ > 1 (unfavorable) (Weber and Chakravorti 1974; Obayomi and Auta 2019). As can be seen from Tables 3 and 4, the values of *R*_L_ were found between 0 and 1, which suggests that the adsorption of AO7 and Cr(VI) onto the prepared modified biochar materials was favorable.

### Desorption and reusability studies

Desorption and regeneration studies of the prepared materials after their saturation by the pollutants are important for the commercial application. The adsorption efficiency of AO7 dye and Cr(VI) by MS-450 and MWC-700 was investigated after five adsorption–desorption cycles (**Fig. S4****(Supplementary Information)**). It was found that the removal efficiency of AO7 dye and Cr(VI) by MS-450 and MWC-700 decreased from 98 to 78% and from 90 to 70%, respectively, after the fifth cycle. In another study, Zaheer et al. (2019) used 0.05 M NaOH for the desorption of AO7 from zero-valent iron nanoparticles (ZvFeNPs). In agreement to the finding of this study, NaOH desorbed AO7 from ZvFeNPs efficiently and the removal efficiency decreased to 82% after five cycles. They stated that slight decrease of removal efficiency after several adsorption–desorption cycles might be related to the formation of hydroxide layer on the adsorbent surface. Similar to the current study, NaOH has been applied for Cr(VI) desorption from microalgal biochar, modified carbon composite, and bio-composite of mango by other researchers (Akram et al. 2017; Daneshvar et al. 2019; Nakagawa et al. 2014). They explained that CrO_4_^2−^ is the dominant form of Cr(VI) in alkaline solution, which can be exchanged by hydroxide (OH^−^). They also described that at high pH solution, electrostatic repulsion due to negatively charged adsorption sites increases desorption of Cr(VI) from adsorbent. The results of the reusability experiment indicated that modified sludge 450 and modified woodchips 700 could be reused successfully for AO7 and Cr(VI) adsorption.

## Conclusions

Magnetic biochar adsorbents were synthesized by the facile co-precipitation method and later evaluated for the AO7 and Cr(VI) removal from water. Adsorption of studied pollutants onto the as-synthesized magnetic biochar materials indicated that the Langmuir isotherm model best fitted to the experimental data. The adsorption process was pH sensitive for both the pollutants (AO7 dye and Cr(VI)) and the optimum pH for highest adsorption was 2. The pseudo-second-order model was fitted well with the experimental data compared with other models. The maximum monolayer adsorption capacities of MS-450 and MWC-700 were observed as 110.27 mg g^−1^ and 80.96 mg g^−1^ for AO7 dye and Cr(VI), respectively. Desorption results showed the reusability of the multiple adsorbents. Therefore, the prepared modified biochar materials could be used as efficient adsorbents for the removal of toxic pollutants from aqueous solution.

## Electronic supplementary material

ESM 1 (DOCX 1027 kb)
