# Supplementary material for: Synthesis and characterization of magnetic biochar adsorbents for the removal of Cr(VI) and Acid orange 7 dye from aqueous solution
Source: Environ Sci Pollut Res Int. 2020 Jun 10;27(26):32874–87. doi: 10.1007/s11356-020-09275-1 (PMC7417418; doi:10.1007/s11356-020-09275-1)
Supplement: Supplementary file 1 — (DOCX 1027 kb) [file 11356_2020_9275_MOESM1_ESM.docx]

**Supplementary Information**

**Synthesis and characterization of magnetic biochar adsorbents for the removal of Cr(VI) and Acid orange 7 dye from aqueous solution**

Chella Santhosh^a^, Ehsan Daneshvar^a*^, Kumud Malika Tripathi^b^, Pranas Baltrėnas^c^, TaeYoung Kim^b^, Edita Baltrėnaitė^c^, Amit Bhatnagar^a^

*^a^ Department of Environmental and Biological Sciences, University of Eastern Finland, P.O. Box 1627, FI-70211, Kuopio, Finland*

^b^ *Department of Bionanotechnology, Gachon University, 1342 Seongnamdaero, Seongnam,*

*13120, South Korea*

^c^ *Institute of Environmental Protection, Vilnius Gediminas Technical University, Saulėtekio al. 11, Vilnius 40, Lithuania*

*Corresponding author: tel.: +358 503696419; e-mail: [ehsan.daneshvar@uef.fi](mailto:ehsan,daneshvar@uef.fi); [ehsandaneshvar_iut@yahoo.com](mailto:ehsandaneshvar_iut@yahoo.com)

**Fig. S1.** Comparative chart of prepared un-modified and modified biochar materials for Cr(VI) and AO7 dye removal (initial concentration: 10 mg L^-1^, pH: 2, adsorbent dosage: 0.5 g L^-1^, contact time: 180 min).

**Fig. S2.** Kinetic studies and modeling of AO7 dye and Cr(VI) removal by modified biochar materials (initial concentration: 10 mg L^-1^, pH: 2, adsorbent dosage: 0.5 g L^-1^, contact time: 180 min).

**Fig. S3.** Isotherm studies and modeling of AO7 dye and Cr(VI) removal by modified biochar materials (initial concentration: 10 mg L^-1^, pH: 2, adsorbent dosage: 0.5 g L^-1^, contact time: 180 min).

**Fig. S4.** Regeneration studies of AO7 dye and Cr(VI) removal by MS-450 and MWC-700, respectively (initial concentration: 10 mg L^-1^, pH: 2, adsorbent dosage: 0.5 g L^-1^, contact time: 180 min and eluent: 0.1 M NaCl).

**Table S1.** Zeta potential of un-modified and modified biochar materials.

| Un-modified biochar samples | Zeta potential (mV) | Modified biochar samples | Zeta potential (mV) |
| --- | --- | --- | --- |
| Sludge 450 | -27.25 | Modified sludge 450 | -45.30 |
| Sludge 700 | -15.15 | Modified sludge 700 | -45.40 |
| Woodchips 450 | -15.62 | Modified woodchips 450 | -27.77 |
| Woodchips 700 | -13.47 | Modified woodchips 700 | -35.94 |
